# A Selective Electrochemical Sensor for Bisphenol A Detection Based on Cadmium (II) (bromophenyl)porphyrin and Gold Nanoparticles

**DOI:** 10.3390/mi15121508

**Published:** 2024-12-18

**Authors:** Fatma Rejab, Nour Elhouda Dardouri, Ahlem Rouis, Mosaab Echabaane, Habib Nasri, Boris Lakard, Hamdi Ben Halima, Nicole Jaffrezic-Renault

**Affiliations:** 1 Faculty of Sciences of Monastir, Laboratory of Advanced Materials and Interfaces (LIMA), University of Monastir, Avenue of the Environment, Monastir 5000, Tunisia; fatmarejab35@gmail.com (F.R.); rouisahlem2@yahoo.fr (A.R.); 2 Faculty of Sciences of Monastir, Laboratory of Physical Chemistry of Materials, University of Monastir, Avenue of Environment, Monastir 5019, Tunisia; nourelhoudadardouri@gmail.com (N.E.D.); hnasri1@gmail.com (H.N.); 3 CRMN, Centre for Research on Microelectronics and Nanotechnology of Sousse, NANOMISENE, LR16CRMN01, Sousse 4054, Tunisia; mosaab.echabaane@gmail.com; 4 Institute of UTINAM, UMR-CNRS 6213, University of Franche-Comté, 16 Gray Road, 25030 Besançon, France; boris.lakard@univ-fcomte.fr (B.L.); nicole.jaffrezic-renault@univ-fcomte.fr (N.J.-R.)

**Keywords:** electrochemical sensor, cadmium metalloporphyrin, square wave voltammetry, bisphenol A

## Abstract

Bisphenol A (BPA) is a commonly synthetic chemical mainly used in producing plastic items. It is an endocrine-disrupting compound that causes irreversible health and environmental damage. Developing a simple method for BPA effective quantitative monitoring is emergently necessary. Herein, a novel electrochemical sensor for BPA detection based on [(5,10,15,20-tetrakis(p-bromophenyl) porphyrinato] cadmium (II) [(CdTBrPP)] and gold nanoparticle (AuNPs)-modified screen-printed carbon electrode (SPCE) was elaborated. CdTBrPP was synthesized and then characterized with Ultraviolet–Visible Spectroscopy (UV/vis), Infrared Spectroscopy (IR), and Proton Nuclear Magnetic Resonance Spectroscopy (^1^H NMR) to confirm its successful synthesis. After drop-coating AuNPs and CdTBrPP on the SPCE, the sensor performance was evaluated using square wave voltammetry (SWV), a linear response in a concentration range from 10^−11^ M to 10^−2^ M, with a low detection limit (LOD) of 9.5 pM. The CdTBrPP/AuNPs/SPCE sensor demonstrates a high selectivity and reproducibility, making it a promising candidate for developing a low-cost water-monitoring system for detecting BPA. Additionally, the proposed sensor effectively detected BPA in both tap and mineral water samples.

## 1. Introduction

Bisphenol A (BPA), 2,2-bis(4-ethylhexyl) propane, has been reported to be a typical endocrine disruptor. It is a hazardous chemical exhibiting estrogenic propriety that can damage the body’s hormone functioning, disrupt the balance of the endocrine system, and negatively impact reproductive health and developmental processes [1,2,3]. The BPA structure is composed of two phenol groups, naturally occurring hormones diethylstilbestrol and estradiol, that generate estrogenic action [4]. Elevated rates of BPA have been associated with several health issues, including cancer, reduced sperm quality, thyroid dysfunction, and increased risk of diabetes [5,6,7,8,9]. In 2023, the European Food Safety Authority established that the Tolerable Daily Intake (TDI) of BPA was 0.2 ng/kg body weight per day [10]. Nevertheless, BPA is widely used in the production of various items, including water bottles, baby feeding bottles, food containers, sports and medical equipment, and thermal papers [11,12]. Unfortunately, BPA leaches from polycarbonate plastics and epoxy resins into the environment, leading to constant exposure for humans and animals. Currently, BPA has been detected in multiple samples, such as clinical matrices, like urine, serum, muscle, and liver tissues, as well as in environmental samples, including water, sewage, sediment, and air.

Therefore, controlling and quantifying BPA is highly important, and it has become one of the most attractive subjects for scientists and researchers. Regarding this need, many methods were used, such as liquid chromatography coupled with electrochemical detection [13], gas chromatography coupled with mass spectrometry [14], enzyme-linked immunosorbent assays [15], and liquid chromatography coupled with mass spectrometry [16]. However, these traditional techniques need costly equipment, present difficult procedures, and are time-consuming. Many studies utilize the electrochemical sensor as an effective and highly sensitive method for BPA detection because of its easy manipulation, short response time, and low cost [17].

Metalloporphyrin, due to its high stability, low toxicity, easy synthesis, and good electron transfer, has become a great candidate for the fabrication of electrochemical sensors. It has a π-conjugated electron system and a central metal, which are utilized as sensitizers and electron mediators [18,19,20], leading to special electrochemical characteristics. Among the metalloporphyrins, we chose a cadmium porphyrin as a sensing material. Cadmium shows a great affinity for porphyrin; it exhibits a large size that can easily fit the coplanarity into the cavity of the porphyrin [21,22,23]. Additionally, porphyrin and cadmium have a rich coordination chemistry [24]. [Cd(Porphyrin)] are less reactive than [Zn(Porphyrin)], and only a few pentacoordinated [Cd(Porphyrin)L] are reported. The only reported Cd-tetravalent porphyrin species is the [Cd(TPP)] complex, TPP being the (5,10,15,20-tetraphenyl)porphyrinato. This complex, functionalized with 4-methoxyphenyl was used in a carbon paste electrode for detecting BPA [25]. In this work, the bromophenyl-functionalized [Cd(TPP)] complex was synthesized and used for the detection of BPA. Metalloprophyrin was associated with different kinds of materials, such as aptamers [26], GO [27], carbon nanotubes [28], and nanoparticles [29], to ameliorate the kinetics of charge transfer and stability during the detection process. Gold nanoparticles (AuNPs) possess a large surface area and high conductivity, making them an excellent option for the development of electrochemical sensors. Their ease of synthesis and manipulation has generated considerable interest in their application [30]. Furthermore, functionalizing gold nanoparticles with organic moieties containing nitrogen offers promising materials for sensor formulations [31]. The metallic properties of AuNPs enhance the conductivity of electrodes, which effectively improves sensing performance [32]. Additionally, the characteristics of gold nanoparticles can be tailored by adjusting their size, shape, and the surrounding chemical environment [33].

In this work, AuNPs were associated with [(5,10,15,20-tetrakis (p-bromophenyl) porphyrinato] cadmium (II) [CdTBrPP] for the modification of a SPCE. CdTBrPP was synthesized and characterized owing to different spectrometries such as ^1^HNMR, UV–vis, and FTIR. For the elaboration of our electrochemical setup, the working electrode SPCE was modified at the first step via drop-casting with AuNPs and the CdTBrPP complex. The electrochemical response of the developed sensor CdTBrPP/AuNPs/SPCE toward BPA and the influencing parameters (pH, scan rate, and interferences) were investigated utilizing CV and SWV.

## 2. Materials and Methods

### 2.1. Materials

In this work, our target molecules are bisphenol, hydroquinone, and catechol. The utilized solvents were N, N-Dimethylformamide (DMF), ethanol, dichloromethane (DCM), and double-distilled water. For the electrochemical experiments, Phosphate Buffer Solution (PBS) served as the electrolyte. All reagents and solvents were sourced from Sigma-Aldrich (Saint Quentin-Fallavier, France), which also provided Gold (III) chloride trihydrate and trisodium citrate dihydrate for the synthesis of gold nanoparticles.

All reagents used in the synthesis of CdTBrPP were commercially obtained from Sigma-Aldrich and were utilized without any additional purification. All procedures were conducted under aerobic conditions. The synthesis of meso-tetra(para-bromophenyl) porphyrin (H_2_TBrPP) was executed following the Adler-Longo method [34].

### 2.2. Apparatus

The techniques used for characterizing the synthesized CdTBrPP are the following ones. The absorption spectra were obtained using a Win ASPECT PLUS UV–visible spectrophotometer (validated for the SPECORD PLUS version 4.2, Germany). Fourier-transform infrared (FT–IR) spectra were recorded with a PerkinElmer Spectrum Two FT–IR spectrometer (Waltham, MA, USA). Proton nuclear magnetic resonance (^1^H NMR) spectra were acquired at room temperature on a Bruker 300 Ultrashield spectrometer (Billerica, MA, USA), with chemical shifts expressed in ppm relative to internal tetramethylsilane (TMS).

The electrochemical measurements were carried out, including screen-printed carbon electrodes (Italsens carbon SPE) purchased from PALMSENS (Houten, The Netherlands). This configuration consisted of a carbon working electrode (WE) (diameter 3 mm), a carbon counter-electrode (CE), and an Ag/AgCl reference electrode (RE). The electrode was submerged in a Phosphate Buffer Solution (PBS) electrolyte (C = 0.1 M, pH = 7). The measurements were conducted with a VMP3 multichannel potentiostat apparatus from Biologic EC-Lab (Seyssinet-Pariset, France), and data acquisition was managed using EC-lab v10.x software.

The Water Contact Angles (WCA) on both modified and unmodified electrodes were assessed using a GBX Scientific Instruments contact angle analyzer (Digidrop Contact Angle Meter paired with Windrop software v1.x, France). A droplet of 3 µL was formed at the end of a syringe needle and positioned onto the sample surface by elevating the sample until contact was made. Contact angles were determined by drawing a tangent near the droplet’s edge, and a minimum of 3 measurements were taken for each sample.

Infrared spectra, used for the characterization of the surfaces of electrodes, were obtained using a Bruker Vertex 70 FTIR spectrometer (from Germany), which was equipped with a DGTS detector and a Platinum ATR accessory that had a diamond crystal. Each sample was scanned 128 times at a resolution of 4 cm^−1^.

The surface morphology of AuNPs and AuNPs/CdTBrPP films was analyzed without any metallization treatment using a high-resolution scanning electron microscope model MIRAN3 TESCAN (form Czech Republic) operating with an electron beam energy of 7 keV.

Mechanical Profilometry: the thickness (T) and arithmetic roughness (Ra) of the films AuNPs and AuNPs/CdTBrPP were assessed using a stylus (2.5 µm)-based mechanical probe profiler Alpha-Step IQ from KLA Tencor (from United States). Measurements for both thickness and roughness were conducted over a scan length of 14,583 µm at a speed of 90 µm/s. The reported values for thickness and roughness represent the averages of at least 3 measurements taken from different locations on the samples.

### 2.3. Synthesis of the Gold Nanoparticles (AuNPs)

The gold nanoparticles were obtained following the method described in the literature [35] by reducing HAuCl_4_·3H_2_O by citrate, with slight modifications. An amount of 1 mM of HAuCl_4_·3H_2_O was mixed with distilled water at a 1:1 ratio in a 250 mL flask, then stirred and heated until it reached boiling. After that, a 38.8 mM solution of a reducing agent (trisodium citrate dihydrate) was promptly added. The mixture was stirred for 20 min, during which the color transformed from pale yellow to dark red and, finally, to wine-red.

### 2.4. Synthesis of [meso-tetrakis(p-bromophenyl) porphyrinato] cadmium (II): [Cd (TBrPP)]

**Figure 1:**
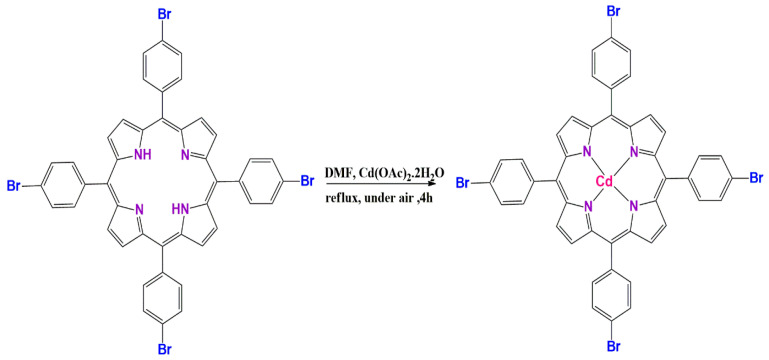
Scheme of the synthesis of [meso-tetrakis(p-bromophenyl) porphyrinato] cadmium (II): [CdTBrPP].

The synthesis reaction of CdTBrPP is illustrated in Figure 1. The free-base porphyrin H_2_TBrPP (400 mg, 0.439 mmol) was dissolved in DMF (90 mL) with an excess of Cd (OAc)_2_·2H_2_O (1 g, 1.344 mmol). The mixture was refluxed under magnetic stirring for 3 h at 140 °C. The evolution of the complexation reaction is followed by thin-layer chromatography (TLC) as well as by UV/Vis spectroscopy by comparing over time the spectrum of the reaction mixture with that of the free-base porphyrin H_2_TBrPP.The mixture was washed with water, and after filtration, the obtained precipitate was dried under vacuum for 2 h to give a green–violet powder with a yield of 75%.

^1^H NMR: (CDCl_3_, 300 MHz): δ(ppm): 8.84 (s, 8 H_β_), 8.15–8.12 (d, 8H, H_o,o′_), 7.77–7.74 (d, 8H, H_m,m′_); UV/Vis (CH_2_Cl_2_): λ_max_ nm (logε): 430(5.34), 565(4.45), 605(3.22); IR cm^−1^: 2995–2853, ν(CH) porphyrin, 998 δ(CCH) porphyrin.

### 2.5. Preparation of CdTBrPP/AuNPs Modified SPCE Electrode

The preparation of the SPCE electrode started by pretreating the electrode using an electrochemical approach. The electrode was submerged in a PBS electrolyte, and a potential of 1.7 V was applied for 180 s, followed by 5 cyclic voltammetry (CV) cycles within a voltage range of 0 to 1.4 V. The obtained electrode was then cleaned with water, dried under nitrogen flow, and finally allowed to dry at ambient temperature.

For the modification of the electrode, a solution of gold nanoparticles (AuNPs) (4 µL, C = 2.8 × 10^12^ particles) was dropped on the pretreated electrode (SPCE) and then dried at room temperature. Subsequently, a solution of CdTBrPP was prepared by sonicating 1.594 mg of this molecule in 200 µL of ethanol, and then a volume of 4 µL from this solution was carefully dropped on the modified AuNPs/SPCE and dried at ambient temperature.

## 3. Results and Discussion

### 3.1. Characterization of CdTBrPP

#### 3.1.1. IR and ^1^H NMR Spectroscopies

**Figure 2:**
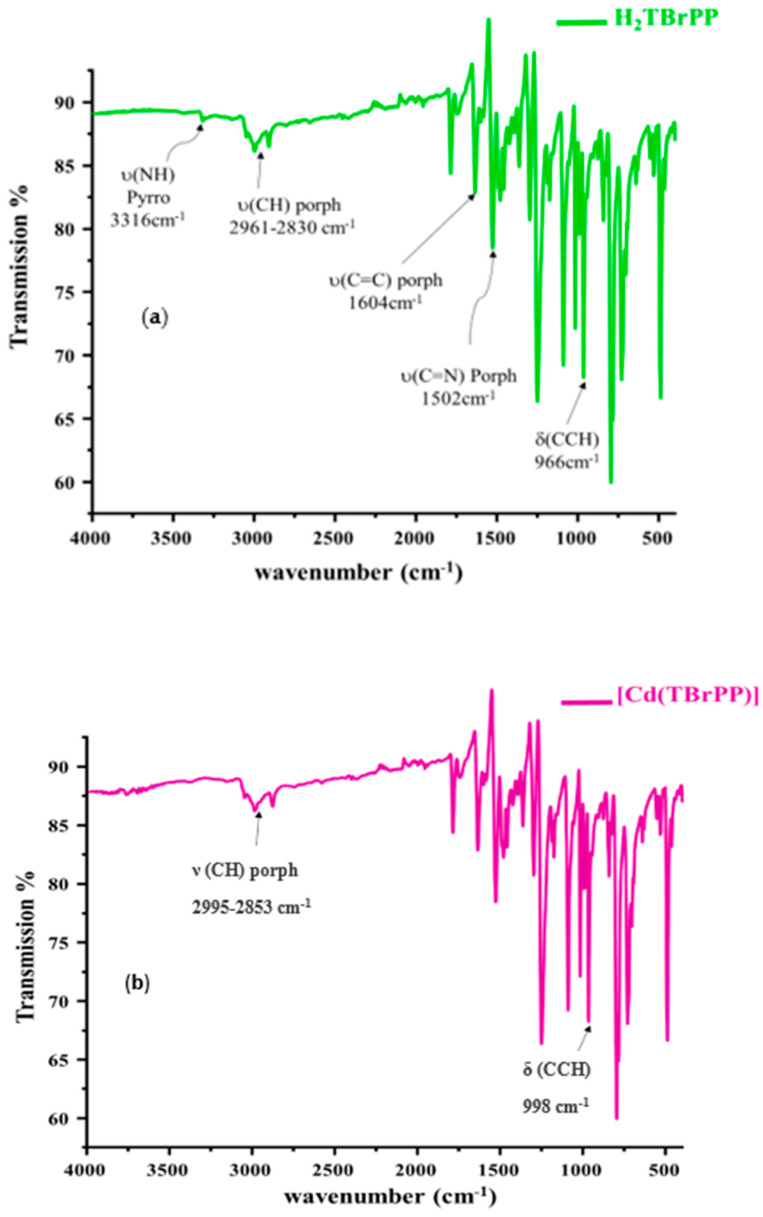
IR spectra (**a**) of the H_2_TBrPP free-base porphyrin (**b**) and the CdTBrPP porphyrin.

The IR spectra of the H_2_TBrPP free-base porphyrin and the [Cd (TBrPP)] are depicted in Figure 2a,b. The H_2_TBrPP exhibits a characteristic IR spectrum of a meso–aryl porphyrin with ν (NH) and ν (CH) stretching frequencies at 3316 cm^−1^ and in the range of 2830–2961 cm^−1^, respectively. The δ (CCH) bending frequency value is 966 cm^−1^. The metalation of the H_2_TBrPP with Cd (II) leads to the disappearance of the absorption band corresponding to the ν (NH) stretching and the shift toward the high fields of the absorption band attributed to δ (CCH) bending from 966 to 998 cm^−1^. These observations confirm the coordination of Cd atoms with the nitrogen of the porphyrin molecules.

**Figure 3:**
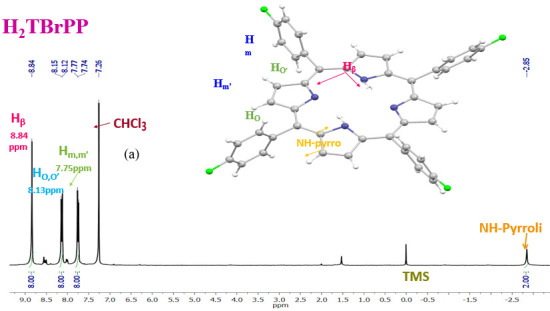

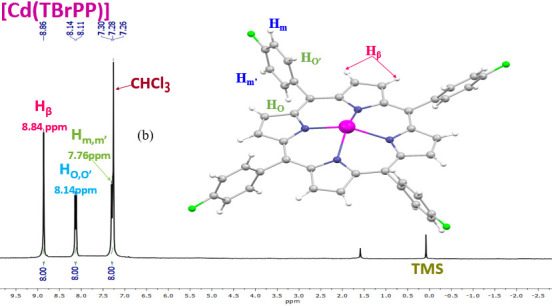
^1^H NMR spectra (**a**) of the H_2_TBrPP free-base porphyrin recorded in CDCl_3_ (**b**) and [meso-tetrakis (p-fluorophenyl) porphyrinato] cadmium (II): [CdTBrPP] recorded in CDCl_3_ with concentration ~10^−3^ M at room temperature.

The ^1^H NMR spectrum of H_2_TBrPP and the [Cd (TBrPP)] is illustrated in Figure 3a,b. As shown in this figure, the chemical shifts of the β-pyrrole protons (H_β_), the phenylic protons (H_o,o′_, H_m,m′_) of the H_2_TBrPP free-base porphyrin, and [Cd(TBrPP)] are very close. Thus, the δ values of H_β_, H_o,o′_, and H_m,m′_ for these two porphyrin species are 8.84/8.13/7.75, 8.84/8.14/7.76 nm, respectively. These values are characteristic of diamagnetic meso–aryl metalloporphyrins with cadmium (II), magnesium (II), and zinc (II) as central metals [36,37,38].

The disappearance of the NH-Pyrrole protons peak at −2.85 ppm for the H_2_TBrPP indicates that the porphyrin is successfully metalated by Cd (II).

#### 3.1.2. UV/Vis Spectrometry

**Figure 4:**
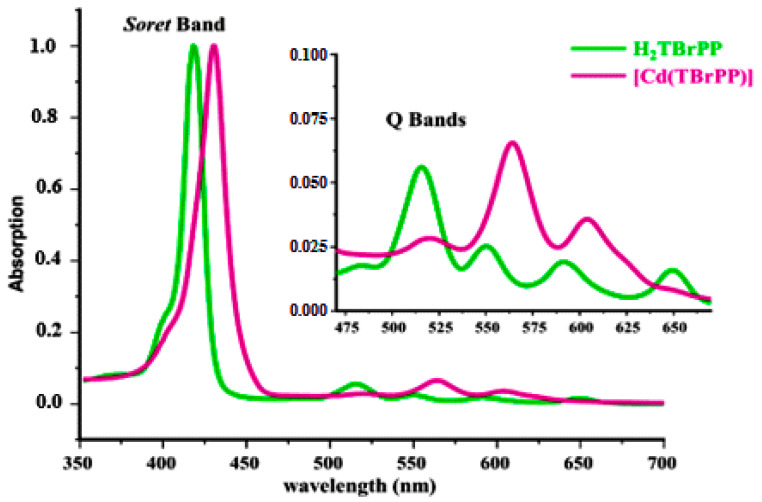
UV/Vis spectra of the H_2_TBrPP free-base porphyrin and the CdTBrPP recorded in dichloromethane at room temperature with concentration ~10^−6^ M.

UV/Vis spectra of the H_2_TBrPP free-base porphyrin and the [Cd (TBrPP)] are depicted in Figure 4. The spectroscopic data of our compounds and some compounds from the literature are assembled in Table 1. As shown by Figure 3, the metalation of H_2_TBrPP porphyrin by Cd (II) leading to the [Cd (TBrPP)] is accompanied by a redshift of the Soret and the Q bands. Thus, the λ_max_ value of the Soret band is 419 nm, while the λ_max_ values of the Q bases values change from 515, 550, 592, and 650 nm to 565 and 605 nm after the porphyrin metalation. The reduction in the number of the Q bands from four to two after the metalation of the porphyrin is due to the change in the symmetry from D_2h_ to D_4h_, respectively.

### 3.2. Characterization of the Modified SPCE

**Figure 5:**
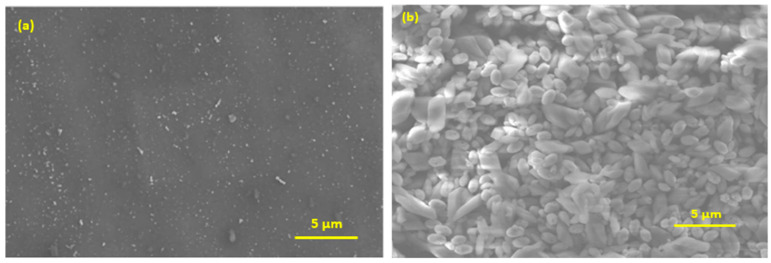
SEM images of the modified electrode (**a**) AuNPs film and (**b**) AuNPs/CdTBrPP film.

Scanning electron microscopy (SEM) was employed to analyze the surface morphology of the AuNPs and AuNPs/CdTBrPP electrodes. Figure 5 shows the surface of AuNPs, which illustrates a uniform film and a smooth morphology. Following the deposition of the CdTBrPP (Figure 5), the structural characteristics and the distribution of the CdTBrPP layer displayed a rough film made up of aggregates. This indicates that the CdTBrPP layer is successfully immobilized onto the AuNPs surface.

The AuNPs film has a thickness of 140 ± 3.51 nm and a roughness of 3.45 ± 0.51 nm. The AuNPs/CdTBrPP film exhibits a thickness of 297.43 ± 3 nm with a roughness of 2.82 ± 0.5 nm.

### 3.3. Water Contact Angle (WCA)

WCA was utilized to evaluate the hydrophilic and hydrophobic properties of the surface of the modified electrodes (Table 2). The WCA of the film of gold nanoparticles was 40, which suggests its hydrophilic propriety. This may be due to the high surface area developed by the AuNPs [41]. After the immobilization of CdTBrPP, there was an increase in the WCA to 87 due to the modification of surface morphology and the high hydrophobicity of porphyrin due to the aromatic rings [42].

### 3.4. Infrared Spectroscopy Characterization

**Figure 6:**
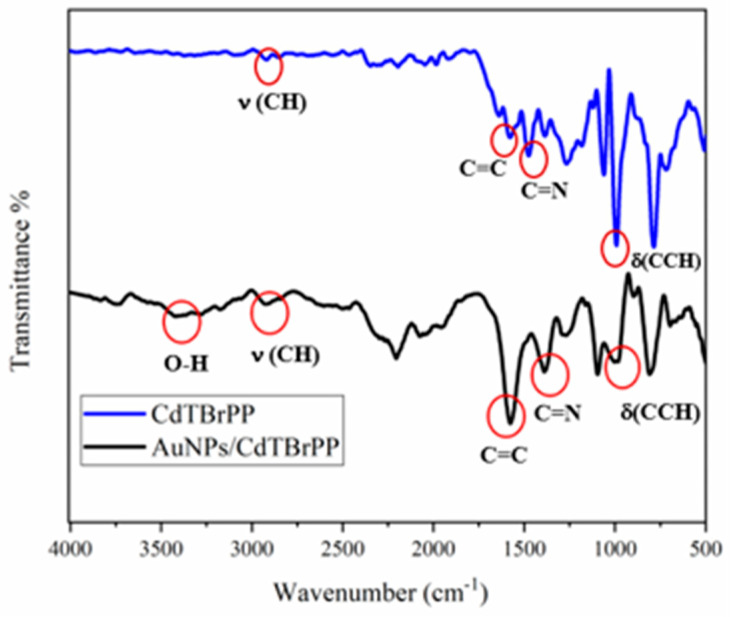
FTIR spectra of the CdTBrPP and AuNPs/CdTBrPP films.

To characterize the surface of the electrochemical sensor, FTIR analysis was carried out. FTIR spectra (Figure 6) revealed that the addition of AuNPs led to the appearance of a new peak at 3421.35 cm^−1^, attributed to the carbonyl and hydroxyl functional groups [43], which supported the successful binding between the porphyrin and the AuNPs. Furthermore, the spectra show shifts in various peaks associated with different functional groups, such as the δ (CCH) stretching frequencies at 1004.56 cm^−1^; the band at 1485.69 cm^−1^ attributed to the ring stretching of C=N band, while the band at 1578.82 cm^−1^ corresponded to the vibration of C=C, and the ν (CH) bending frequency at 2928.8 cm^−1^. These results confirm the successful immobilization of our sensing platform.

### 3.5. Electrochemical Characterization of the SPCE Modified Electrodes

The electrochemical characteristics of the bare SPCE, CdTBrPP/SPCE, and Cd TBrPP/AuNPs/SPCE were examined through cyclic voltammetry (CV) in PBS buffer (C = 0.1 M) containing 10 mM [Fe (CN)_6_]^−2/3^, with a scan rate of 50 mV/s (Figure 5).

**Figure 7:**
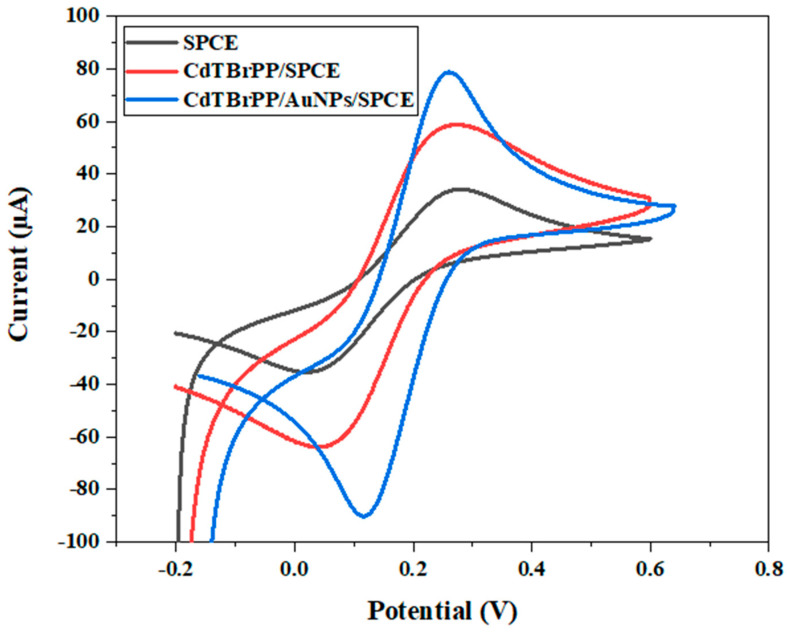
Cyclic voltammograms of bare SPCE electrode CdTBrPP/SPCE and CdTBrPP/AuNPs/SPCE in a PBS solution of 10 mM [Fe (CN)_6_]^−2/−3^ with a scan rate of 50 mV·s^−1^.

Figure 7 illustrates that the bare SPCE exhibits lower oxidation and reduction peak intensities located at 0.28 V and 0.02 V with a peak separation order of 0.26 V. After the immobilization of CdTBrPP, we denoted an increase in both oxidation and reduction peak intensities accompanied by a shift of the peak maxima position respectively 0.22 V and 0.03 V with a peak separation (ΔE) of 0.19 V. This indicates a quasi-reversible electrochemical behavior for the modified electrode [44] highlighting the role of CdTBrPP in improving the charge transfer process.

The modified CdTBrPP/AuNPs/SPCE demonstrates the highest cathodic and anodic peak currents compared with the other electrodes. These peaks are located at 0.22 V and 0.09 V with the lowest ΔE of 0.14 V. This indicates the quasi-reversibility of the electrode CdTBrPP/AuNPs/SPCE, which may be explained by the presence of AuNPs that enhance the conductivity of the electrode surface, facilitating the charge transfer process [45]. Consequently, the modified electrode CdTBrPP/AuNPs/SPCE is an excellent sensing platform.

**Figure 8:**
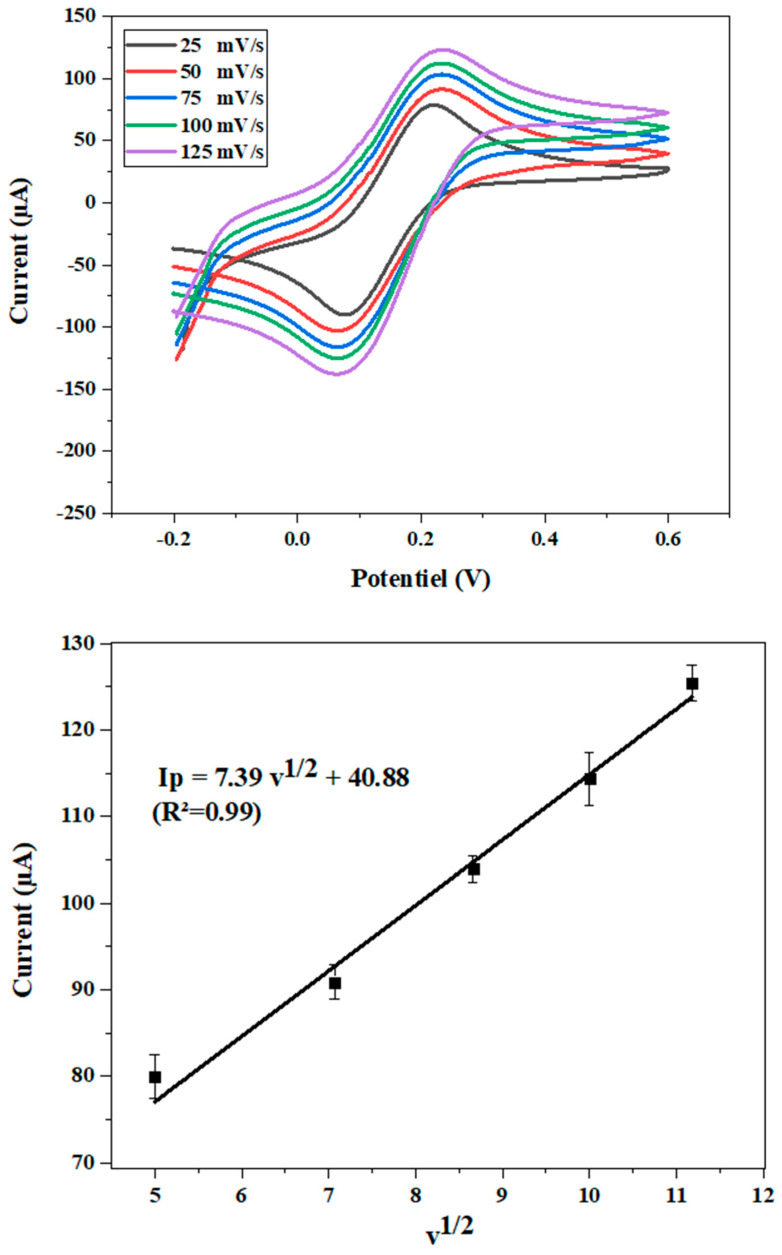
Plot of current versus square root of scan rate (v^1/2^).

To identify the active surface area of the modified electrode CdTBrPP/AuNPs/SPCE, the magnitude of the peak cathodic current Ip was traced at different scan rates ranging from 25 to 125 (Figure 8). It was determined using the Randles–Sevcik equation [45]:

I_p_ = 2.69 × 10^5^ × A × D^(1/2)^ × n^(3/2)^ × v^(1/2)^ × C (1)

where n = 1 is the number of electrons involved in the redox reaction; C is the concentration of [Fe (CN)_6_]^−2/−3^; A is the area of the electrode (cm^2^), and D is the diffusion coefficient of the analyte. For [Fe (CN)_6_]^−2/−3^, the measured diffusion constant is 7.6 × 10^−6^ cm^2^ s^−1^ [46]. From the slope of the I_p_ vs. v^1/2^ graph, I_p_ = 7.39 v^1/2^ + 40.88, the calculated surface area of CdTBrPP/AuNPs/SPCE was determined to be 9.90 mm^2^, the geometrical surface area being 7.00 mm^2^. The factor of 1.4 was brought by the CdTBrPP/AuNPs modification.

### 3.6. The Electrochemical Behavior of BPA on Different Electrodes

The electrochemical response of AuNPs/SPCE, CdTBrPP/SPCE, and CdTBrPP/AuNPs/SPCE electrodes toward the bisphenol A molecule was evaluated using cyclic voltammetry. It was measured in PBS buffer (C = 0.1 M, pH = 7) with a scan rate of 50 mV/s in a potential range from −0.6 to 1 V after addition of 1 µM BPA.

**Figure 9:**
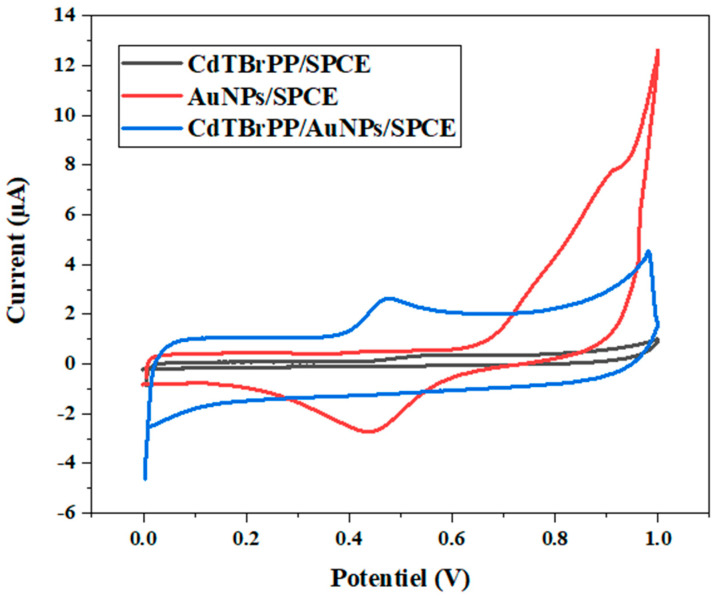
Electrochemical behavior of AuNPs/SPCE, CdTBrPP/SPCE, and AuNPs/CdTBrPP/SPCE in PBS buffer (C = 0.1 M, pH = 7) with the presence of 1 µM BPA.

Figure 9 shows that the oxidation peak of BPA is absent for modified electrode AuNPs/SPCE, whereas it appears in both CdTBrPP/SPCE and CdTBrPP/AuNPs/SPCE electrodes. This is in good agreement with the high affinity of CdTBrPP toward BPA. In addition, the oxidation peak intensity of BPA is higher for CdTBrPP/AuNPs/SPCE compared with CdTBrPP/SPCE. This confirms the important role of AuNPs in enhancing the charge transfer process of the developed sensor.

### 3.7. Optimization of Experimental Conditions

#### 3.7.1. pH Effect

**Figure 10:**
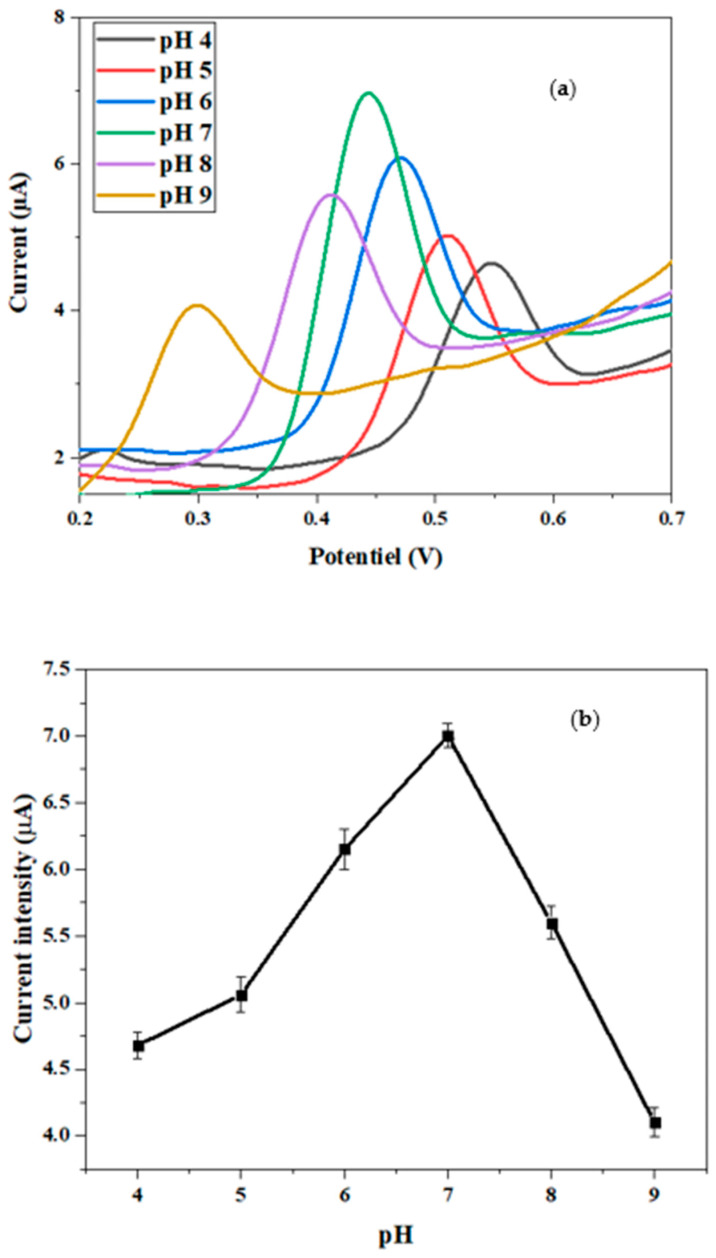

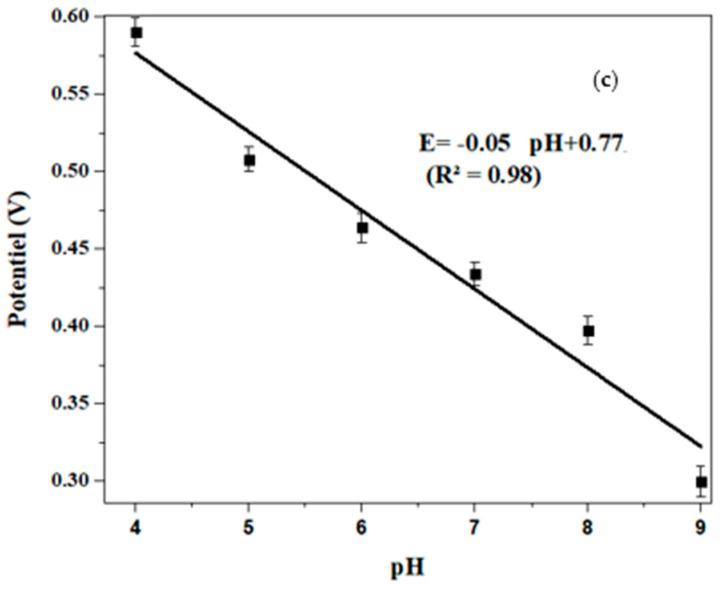
(**a**) CV of 10^−2^ M BPA at CdTBrPP/AuNPs/SPCE in 0.1 M PBS with different pH values from 4 to 9; (**b**,**c**) variation in peak current and peak potential versus pH.

The pH is an imperative parameter influencing the performance of the response of the designed sensor toward BPA. The effect of this parameter was studied using CV in PBS buffer containing 10 µM of BPA with a scan rate of 50 mV/s, in the pH range from 4 to 9 (Figure 10a). As resumed in Figure 10b, within the pH range of 4 to 7, the peak current increased, which proves that the charge transfer was higher [47]. On the other hand, in the pH range of 7 to 9, the peak current decreased, likely due to reduced sensor activity at higher pH levels. As a result, the pH of 7 was chosen as the ideal pH value for our sensor to obtain the highest sensitivity.

To clarify the charge transfer mechanism during the detection of BPA, using our sensor, the oxidation peak potential (E) was plotted versus the BPA solution pH within the selected pH range (Figure 10c). It shows that the oxidation peak potential (E) was inversely proportional as a function of the electrolyte pH, following the linear relationship:

E = −0.05 pH + 0.77 (R^2^ = 0.98) (2)

with a plot slope of 52 mV pH^−1^, which is approximately the theoretical value of 57.6 mV/pH [25]. This suggests that the numbers of electrons and protons transferred during the electrochemical oxidation of BPA are equal.

#### 3.7.2. Scan Rate

**Figure 11:**
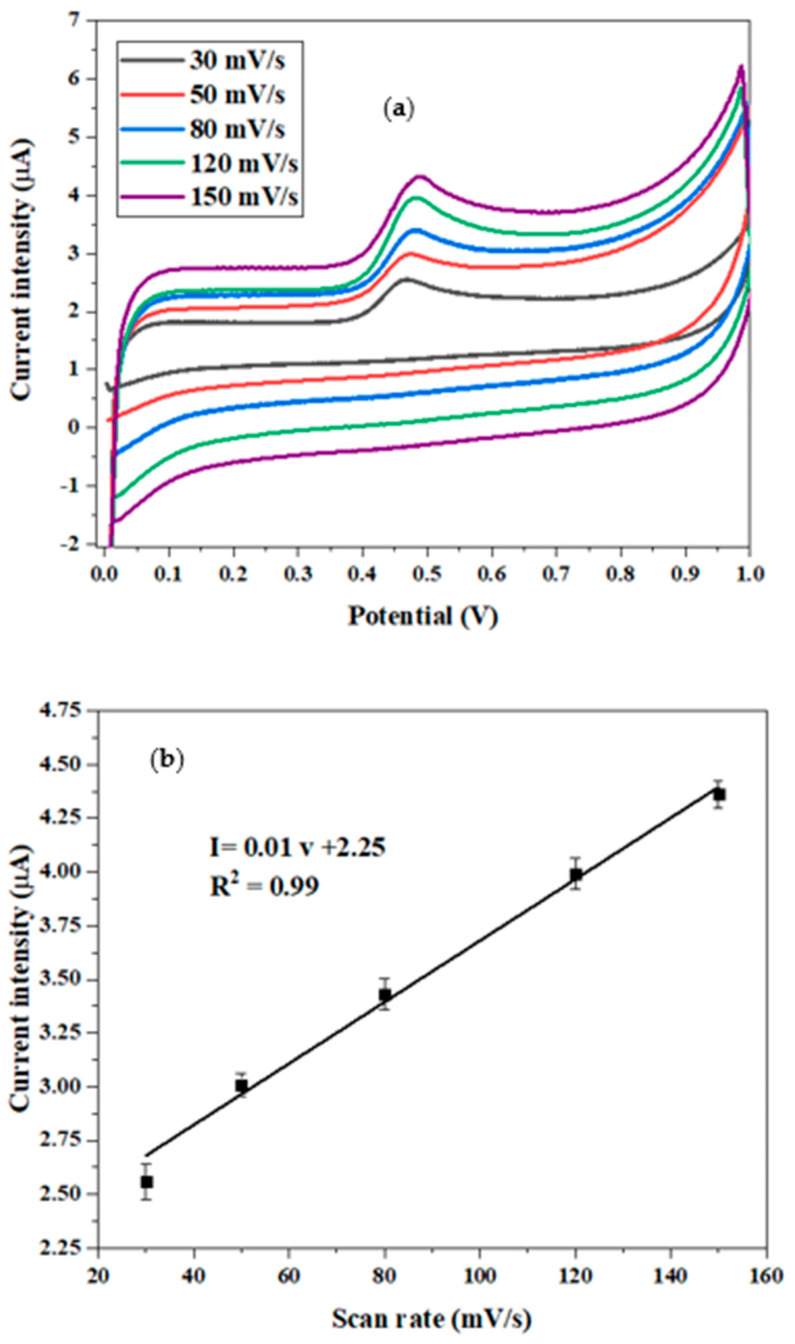

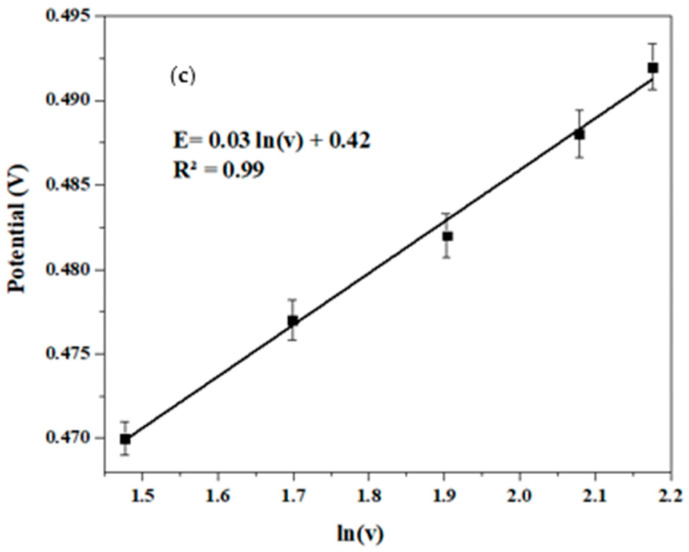
(**a**) Cyclic voltammograms of 10^−2^ M BPA at CdTBrPP/AuNPs/SPCE at various scan rates (30–150 mV·s^−1^) in 0.1 M PBS (pH = 7); (**b**) plot of peak current vs. scan rate; (**c**) plot of E vs. ln (scan rate).

The effect of the scan rate (v) on the peak current of the oxidation of BPA was explored in a PBS electrolyte buffer (C = 0.1 M, pH = 7) in the presence of 10 µM of bisphenol A, using cyclic voltammetry (CV) in the potential range from 0 to 1 V (Figure 11a). As shown in Figure 11b, the current peak exhibits a linear increase with the scan rate (v) in the range of [30–150] mV/s following the equation I = 0.01v + 2.25 (R^2^ = 0.99). This behavior indicates that the electrochemical detection of BPA is governed by an adsorption-controlled process on the CdTBrPP/AuNPs/SPCE.

**Figure 12:**
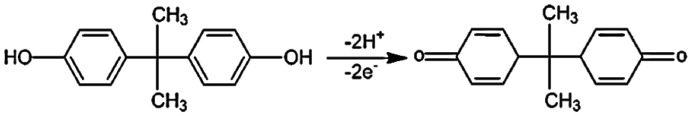
Electrooxidation mechanism of BPA.

Figure 11c highlights a linear relationship between the peak potential (E) and the decimal logarithm of the scan rate. This relationship can be described by the equation E = 0.03lnv + 0.42 (R^2^ = 0.99), which is for an adsorption-controlled process known to follow the Laviron equation [25].

(3) $$E=E^{0}+\frac{RT}{\alpha nF}\times \ln\left(\frac{RTK^{0}}{\alpha nF}\right)+\left(\frac{RT}{\alpha nF}\right)\times ln(v)$$

where α is the transfer coefficient typically recognized as 0.5 in the case of the irreversible process [48]; K^0^ is the standard rate constant of the reaction; n is the electron transfer number involved in the rate-determining step; v is the scan rate; E^0^ is the formal redox potential; R is the gas constant; T is the absolute temperature, and F is Faraday’s constant. The value of αn is determined from the slope of E vs. lnv, which is found around 0.96, which indicates that the number of electrons transferred (n) is close to two. As a result, the electrooxidation process of the BPA on the CdTBrPP/AuNPs/SPCE requires two electrons and two protons, as demonstrated in Figure 12. According to other research, the high affinity between BPA and the metalloporphyrin CdTBrPP can be explained by the specific coordination of the central cadmium ions and the hydroxyl group of BPA [49,50], and π–π interactions between the porphyrin ring and the aromatic structure of BPA can enhance the selectivity [51].

### 3.8. Analytical Performance of the Proposed Sensor

**Figure 13:**
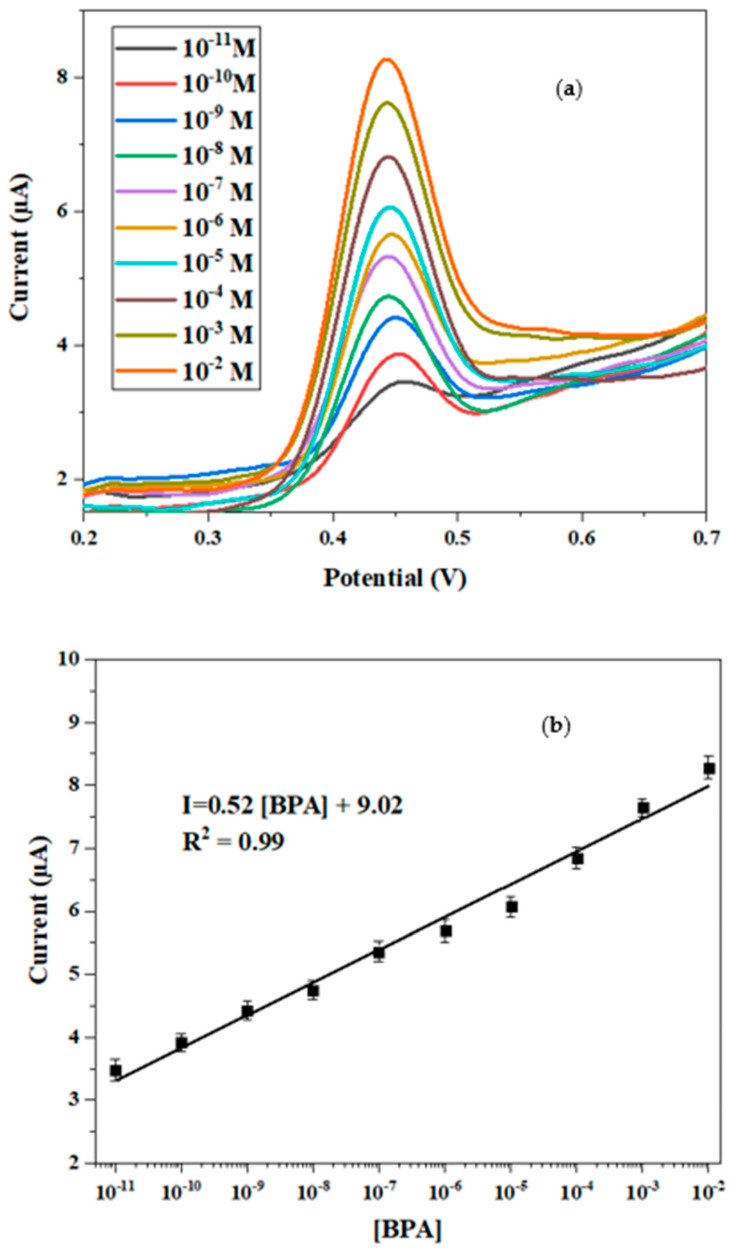
(**a**) SWV response of CdTBrPP/AuNPs/SPCE at various concentrations of BPA in PBS (C = 0.1 M, pH = 7); (**b**) calibration plot of peak current versus log [BPA].

The calibration curve of the proposed sensor was obtained from the electrochemical response of CdTBrPP/AuNPs/SPCE toward various concentrations of bisphenol A ranging from 10^−11^ to 10^−2^ M. It was investigated using SWV in a voltage range from 0.1 V to 0.7 V in PBS buffer (C = 0.1 M, pH = 7) (Figure 13). As shown in Figure 13a,b, the peak current corresponding to the potential 0.45 V gradually increases when the concentration increases. The peak current follows a linear relationship with the logarithm of the concentration expressed by the following equation:

I = 0.52 × log [BPA] + 9.02 (R^2^ = 0.99)

This indicates that the developed sensor exhibits a high sensitivity toward BPA of 0.52. The limit of detection was determined based on the following formula [52]: LOD = 3s/a, where s is the standard deviation at low concentration, and a is the slope of the calibration curve.

The calculated LOD was found to be 9.5 pM. These results demonstrate that the proposed sensor presents a detection limit in the lower range and a wide detection range compared to published BPA electrochemical sensors (Table 3).

#### 3.8.1. Reproducibility, Repeatability, and Stability

The reproducibility of our proposed sensor was studied using three identical electrodes for the determination of 1 µM of BPA using SWV with the same voltage range as the precedent paragraph. The finding demonstrated a low relative standard deviation order of 2.78%. Additionally, the repeatability was estimated by repeating the last measure of each electrode three times. The given result indicates an RSD order of 1.68%. These results confirm that our sensor has excellent reproducibility and repeatability. Furthermore, the stability of the developed sensor was assessed by storing the electrode at room temperature for 5 days and then testing its response toward 1 µL of BPA. The sensor retained 87% of its initial response, indicating that the CdTBrPP/AuNPs/SPCE exhibited excellent storage stability.

#### 3.8.2. Selectivity

Selectivity study is a crucial performance of a sensor; it evaluates its ability to determine the target analyte in a solution containing a potential interfering species. The selectivity of the CdTBrPP/AuNPs/SPCE electrode toward the bisphenol A was tested by adding to a 1 µM of this target other potential interferents at concentrations that were 100 times higher than BPA.

**Figure 14:**
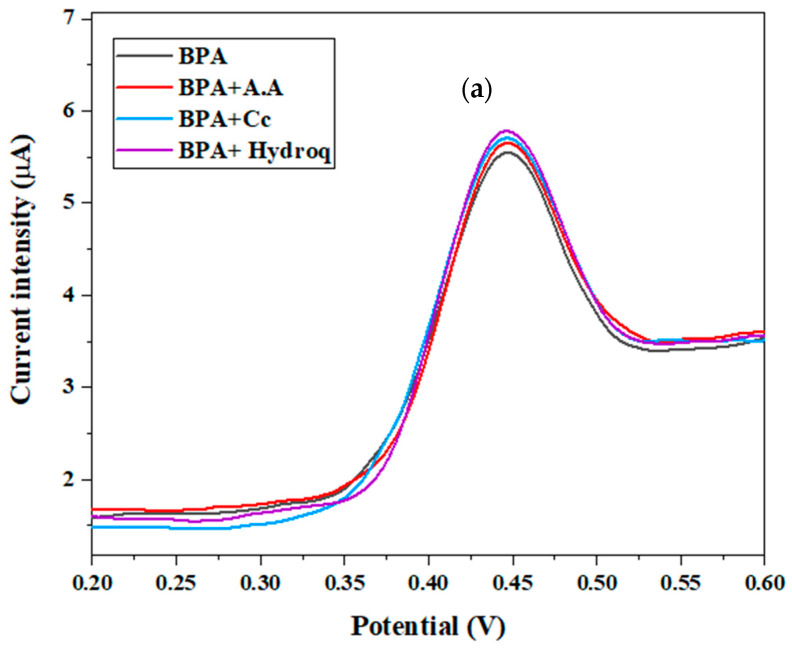

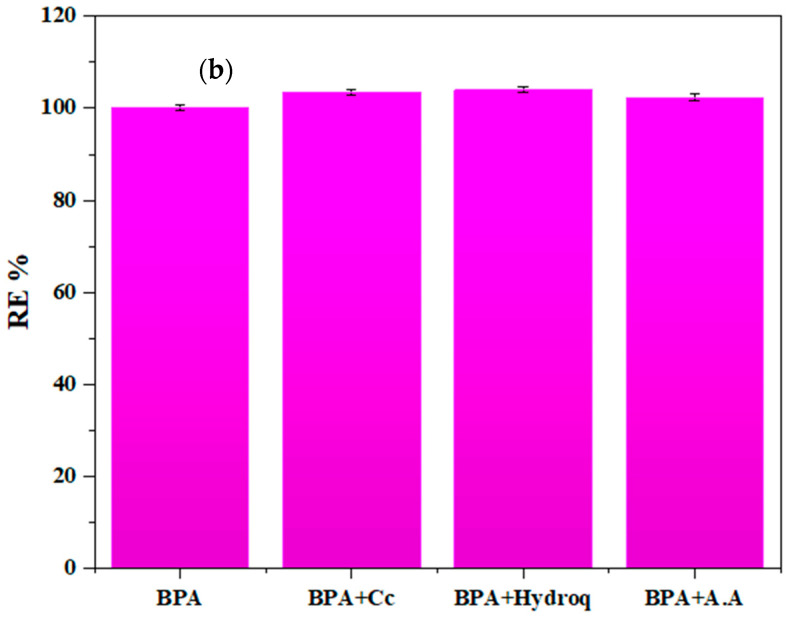
Influence of interferents, 100-fold concentration, on the electrode response towards 1 µM BPA (catechol, hydroquinone, ascorbic acid) (**a**) the voltammogram; (**b**) the histogram.

The observed signal change was less than 5% in the presence of these different interfering substances (as presented in Figure 14). This high level of selectivity confirms that the developed sensor can specifically detect bisphenol A in different sample matrices, making it suitable for real sample applications.

### 3.9. Real Sample Analysis

The usefulness of the proposed sensor, CdTBrPP/AuNPs/SPCE, was evaluated by detecting BPA in tap and mineral water samples. Under optimal conditions, 4 mL of each sample was added to 36 mL of PBS (C = 0.1 M, pH 7). BPA was not detected in either sample. Subsequently, the samples were spiked with various concentrations of BPA and analyzed. As presented in Table 4, the average recovery rates were 103.9–105.7% and 104.6–106% for tap and mineral water, respectively. This demonstrates that the sensor is well-suited for use in natural samples.

## 4. Conclusions

A flexible and low-cost electrochemical sensor was developed based on novel Cd-metalloporphyrin CdTBrPP and gold nanoparticles modified SPCE. It presents a high sensitivity for BPA determination. The sensing label CdTBrPP/AuNPs/SPCE was characterized using different analytical methods such as Cyclic voltammetry (CV), Scanning Electron Microscopy (SEM), Infrared Spectroscopy Characterization (FTIR), Water Contact Angles (WCA), and Mechanical Profilometry. These techniques confirm the successful immobilization of AuNPs and CdTBrPP. The fabricated BPA sensor exhibits good linearity over a wide detection range (10^−11^ M to 10^−2^ M) with high sensitivity and a very low limit of detection order to 9.5 × 10^−12^ M. In addition, the fabricated sensor shows satisfactory reproducibility, repeatability, and stability. Moreover, the proposed sensor has a good selectivity toward PBA and shows interesting results for real mineral and tap water applications with a recovery rate of around 105%. Therefore, it can be reasonably concluded that our sensor is a very promising and effective platform for monitoring this toxic agent in various environmental or food samples.

## Figures and Tables

**Table 1:** UV/Vis data of H_2_TBrPP and a selection of cadmium (II) metalloporphyrins spectra were recorded in dichloromethane at room temperature.

**Compound**	**Soret Band**	**Q Bands**	**Ref.**
**λ_max_ in nm (log ε)**			
H_2_TMPP ^a,d^	420 (5.49)	517 (4.20), 553 (3.96), 589 (3.75), 645 (3.61)	[39]
H_2_TPP ^b,c^	418 (7.96)	515 (6.60), 549 (6.30), 591 (6.27), 647 (6.27)	[40]
H_2_TBrPP ^b^	419 (5.45)	515 (4.21), 550 (3.93), 592 (3.75), 650 (3.58)	This work
[Cd (TMPP)] ^b^	433 (5.47)	568 (4.43), 609 (3.18)	[39]
[Cd(TBrPP)] ^b^	430 (5.34)	565 (4.45), 605 (3.22)	This work

^a^: λ_max_ (nm), ^b^: All data are from spectra recorded in dichloromethane, ^c^: TPP = meso–tetraphenylporphyrinate, ^d^: TMPP = meso–tetrametoxyphenylporphyrinate.

**Table 2:** WCA on the AuNPs and AuNPs/CdTBrPP films.

**Samples**	**AuNPs**	**AuNPs/CdTBrPP**
	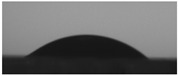	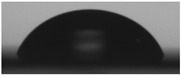
WCA (°)	37.9 ± 3.7	70.56 ± 1.56

**Table 3:** Comparison of the prepared sensor for the detection of BPA with other published sensors.

Electrode Material	Technique	Range	LOD	
[Cd(TMPP)]/CPE	CV	1.5 nM–15 µM	13.5 pM	[25]
NiTPP/carbon nanotubes	Amperometry	0–60 µM	98 nM	[48]
(Fe(III)TMPP)-TRGO/AuE	EIS ^a^	10^−12^–10^−9^ M	0.2 pM	[52]
TiO_2_-SWCNTs/HMH/CPE	DPV ^b^	3.0 nM–450 µM	1.0 nM	[53]
10%Pt@Ti_3_ C_2_ Tx/GCE	DPV	50 nM–5 μM	32 nM	[54]
CeO_2_/Co_3_O_4_–Fe_2_O_3_@CC	DPV	0.5–30 μM	8.7 nM	[55]
NiO/ZnO/rGO/PtE	SWV	0.07–30 μM	4.0 nM	[56]
AuSiO2700/CHI/Pt	DPV	1–1000 nmol L^–1^	7.75 nM	[57]
CdTBrPP/AuNPs/SPCE	SWV	10^−11^–10^−2^ M	9.5 pM	**This work**

^a^ Electrochemical Impedance Spectroscopy. ^b^ Differential Pulse Potential.

**Table 4:** Bisphenol A monitoring with the proposed sensor in a real sample test.

	Added (M)	Found (M)	Recovery Rate (%)	RSD (%) (n = 3)
**Tap water**	10^−3^	1.039 × 10^−3^	103.9	1.56
	10^−9^	1.057 × 10^−9^	105.7	1.24
**Mineral water**	10^−3^	1.046 × 10^−3^	104.6	2.11
	10^−9^	1.060 × 10^−9^	106.0	1.15

## Data Availability

The data that support the findings of this study are available from the corresponding author upon reasonable request.
